# The Present-Day Treatment of Syphilis

**Published:** 1900-04-14

**Authors:** 


### THE PRESENT-DAY TREATMENT OF SYPHILIS.

Speaking recently at the London Polyclinic on the Present-day Treatment of Syphilis, Mr. Jonathan Hutchinson pointed out in the first instance that the mortality from this disease had been considerably reduced in recent years, and that this was in accord with what all observers had noticed as to the milder character of the disease at the present time. With regard to treatment, he maintained that mercury had gained the day, being now almost universally used. Yet it was not absolutely essential, since syphilis, like other specific fevers, might run its course and die out; or, in other words, there might be spontaneous recovery without treatment. The advantages of giving mercury, however, were considerable. If given early enough, it might prevent the occurrence of any secondary symptoms, and if it did not prevent them it would render them milder. As to the way in which mercury was useful, Mr. Hutchinson suggested that it was antagonistic to the low organisms on which the disease depends. We would point out, however, that the recognition of the utility of mercury by no means depends on an acceptance of this theory of its *modus operandi*. In regard to the details of treatment, the first question was whether an early chancre should be removed. Such removal, if it could be done early enough, had, he said, a good basis of common-sense, but there were practical difficulties in the way, due on the one hand to the fact that the case was not usually seen early enough, and on the other to the insidious manner in which the virus got into the system. If, however, the patient were seen within a week, or within a fortnight of being exposed to the contagion, removal might be effectual, and even if not, it might render the future manifestations milder. For such removal Mr. Hutchinson used a Paquelin's cautery, and in any case as a local application he thought black wash was the best. Granting, then, that mercury was to be employed, the question came, When should it be commenced? To this Mr. Hutchinson answered, "The earlier the better." We should not wait for the appearance of secondary symptoms, but should give mercury with the definite object of suppressing the disease altogether, and his experience was, that if it were given from the fourth or the fifth week after infection, and persevered in long enough no further symptoms appeared. As to the method of administering mercury, his own plan was to give it continuously for long periods without interruptions. He had found that if given for a year and then discontinued, an eruption might appear, but it would be mild; and if mercury were given again it would soon vanish. In regard to the form in which it should be administered, there was a good deal of difference of opinion. Many used inunction or injection, but he preferred to introduce it by the mouth. He gave one grain of grey powder in pills three times a day for a week, then four times a day, and at the end of a fortnight five times a day, combining it with opium in quarter-grain doses to prevent diarrhœa. He directed his patients to keep warmly clad, and to go on for a year with this treatment and to fear no evil effects. During the course of the treatment, either iron or quinine might be given without in any way interfering with the pills. Tertiary syphilis might require iodide of potassium, but this also should be given in a separate mixture and not combined, as was so often done, with any mercurial.

